# Mean corpuscular haemoglobin concentration (MCHC): a new biomarker for high-altitude pulmonary edema in the Ecuadorian Andes

**DOI:** 10.1038/s41598-022-25040-5

**Published:** 2022-12-01

**Authors:** Karen Sánchez, Lenin Ramírez-Cando, Wilfre Machado, Anita Villafuerte, Santiago Ballaz

**Affiliations:** 1 School of Biological Sciences and Engineering, Yachay University for Experimental Technology and Research (Yachay Tech), Hacienda San José S/N, Proyecto Yachay, 100115 Urcuquí, Ecuador; 2 School of Mathematical and Computational Sciences, Yachay University for Experimental Technology and Research (Yachay Tech), Urcuquí, Ecuador; 3 Hospital Claudio Benati (HCB), Zumbahua, Ecuador; 4 Medical School, Universidad Espíritu Santo, Samborondón, Ecuador

**Keywords:** Biological techniques, Biomarkers

## Abstract

Ascent to high altitude (> 3000 m height above sea level or m.a.s.l) exposes people to hypobaric atmospheric pressure and hypoxemia, which provokes *mountain sickness* and whose symptoms vary from the mild acute mountain sickness to the life-threatening, high-altitude pulmonary edema (HAPE). This study analysed the risk factors underlying HAPE in dwellers and travellers of the Ecuadorian Andes after sojourning over 3000 m height. A group of HAPE patients (N = 58) was compared to a NO HAPE group (N = 713), through demographic (ethnicity, sex, and age), red blood cell parameters (erythrocytes counts, hematocrit, median corpuscular volume, median corpuscular haemoglobin, and median corpuscular haemoglobin concentration (MCHC)), altitude (threshold: 3000 m.a.s.l.), and health status (vital signs) variables. Analysis of Deviance for Generalised Linear Model Fits (logit regression) revealed patterns of significant associations. High-altitude dwellers, particularly children and elder people, were HAPE-prone, while women were more tolerant of HAPE than men. Interestingly, HAPE prevalence was strongly related to an increment of MCH. The residence at middle altitude was inversely related to the odds of suffering HAPE. Ethnicity did not have a significant influence in HAPE susceptibility. Elevated MCHC emerges like a blood adaptation of Andean highlanders to high altitude and biomarker of HAPE risk.

## Introduction

The term high-altitude illness or simply *mountain sickness*, is commonly used to describe a series of cerebral and pulmonary syndromes that develop shortly after rapid ascent above 2500 m, where the atmospheric and inspired partial O_2_ pressures drop from 160 mmHg down to 118 mmHg^1^. The significant reduction of the partial O_2_ atmospheric pressure leads to its poor diffusion from the alveolar air into the blood, while the percentage of O_2_ carried out by haemoglobin drops down below 90% (hypoxemia). Under such hypobaric atmospheric conditions, the human body reacts by a series of mechanisms, known as acclimatisation, to compensate for the desaturation of haemoglobin. The acclimatisation process takes from hours to days, and even weeks^2^.

A failure in acclimatization causes *mountain sickness*, an overall term to name several high altitude illnesses ranging from the benign, self-limiting acute mountain sickness to more serious clinical manifestations like the high-altitude pulmonary edema or HAPE ^1^ and eventually the high-altitude cerebral edema^3^. HAPE is a life-threatening condition produced by a rapid accumulation of extravascular fluid flooding the pulmonary alveoli^4,5^. Unfortunately, the precise pathophysiology of HAPE is not well understood^6–9^. It is believed that an exaggerated increase in the hypoxic pulmonary artery and pulmonary capillary pressure in conjunction with an uneven hypoxic pulmonary vasoconstriction are pivotal in its pathogenesis^7,10,11^. Antecedents of high-altitude related pulmonary illnesses increase the risk for HAPE^10,12–14^, thus pointing to genetic factors^15–17^ and the adaptation to high altitude. It was herein investigated risk factors for developing HAPE in an elevation of 3000 m above sea level (m.a.s.l.) in the Ecuadorian Andes, where atmospheric pressure decreases to levels of 525 mmHg.

## Results

### Survey of distribution

From a total of 771 patients who had been studied (age average: 32.54 ± 23.2 years) including 332 women and 439 men, 58 patients were admitted in the emergency room of the Claudio Benatí Hospital (3508 m.a.s.l.) with evident symptoms of HAPE after sojourning at 3500–4000 m.a.s.l. in Zumbahua. The age average of the HAPE group (13 women and 45 men) was 34.6 ± 27 years. Within the data frame, a total of 448 people were self-perceived as indigenous and 323 as *mestizos* from Ecuador. There were no Europeans among the subjects analysed. All data sampled at 95% confidence level has a margin of error of 5.3%.

### Analysis of HAPE risk factors

**Figure 1:**
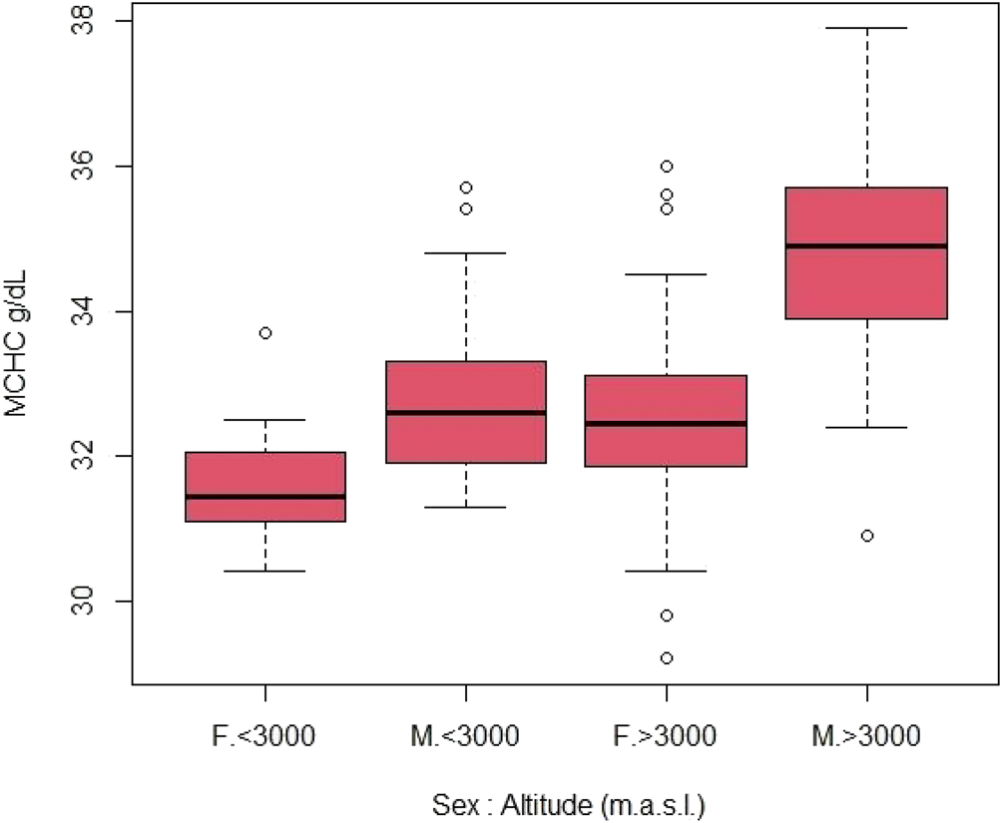
Differences in MCHC by sex and altitude of residence.

**Figure 2:**
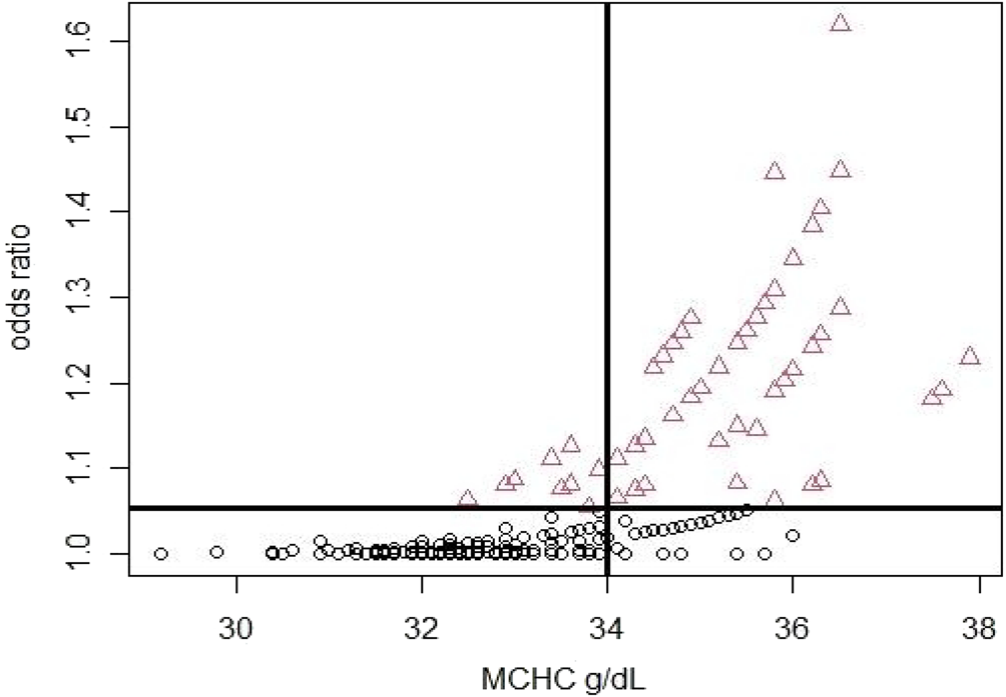
Odds ratio and its relationship with MCHC in the cohorts analysed. Red triangles represent odds ratio ≥ 1.05, while black circles denote odds ratio < 1.05.

Demographic factors like sex and age, altitude of permanent residence (> 3000 m.a.s.l.)*,* and the MCHC blood parameter was significantly associated with HAPE susceptibility (see Figs. 1, 2 and Table 1).

**Table 1:** Analysis of deviance for the GLM model fitted.

Variable	*df*	Deviance	Residual df	Residual deviance	*p* value (> chisqrt)
Null			231	94.452	
Sex	1	7.5273	230	86.925	0.0061
MCHC (g/dL)	1	10.0405	229	76.884	0.0015
Altitude (m.a.s.l.)	1	2.8498	228	74.035	0.0913
Age (years)	3	6.4774	225	67.557	0.0905

Figure 1 represents the difference due to sex and altitude. MCHC in males living over 3000 m.a.s.l. is higher by 7% compared to the sample mean (N = 771). In contrast, in females, MCHC differences across altitudes did not reach statistical significance. Also according to the General Linear Model or GLM, which generalizes linear regression by allowing the linear model to be related to the response variable via a *link function* and by allowing the magnitude of the variance of each measurement to be a function of its predicted value, the rest of the factors analysed (ethnicity, RBCs, HCT, HGB; MCV, MCH; blood pressure, heart rhythm, breathing rate, and blood O_2_ saturation), turned out irrelevant to HAPE susceptibility (data not shown).

In Eq. 2 (shown below), the intercept for the model was settled with SEX_Female_, MCHC (continuous), ALTITUDE_<3000_ and AGE_25–64_ as starting points; odds linked to this combination of factors was ≈ 1e^−19^. These conditions pointed to the lowest odds for the cohort under analysis. Conversely, the following subset of conditions: being male, over 65 years old, with MCHC = 37 and residing > 3000 m.a.s.l. in the Andean highlands, led to odds of ≈ 1.89, that is an increase of risk compared with the base condition of the model (SEX_Female_, ALTITUDE_<3000_ and AGE_25–65_). The above was graphically represented in Fig. 2 as a function of MCHC.

Equation 2. Logit model, representing the logarithmic odds associated with HAPE condition. In the case of categorical variables, one of the levels of any categorical variable is chosen as a reference to construct the intercept (SEX_Female_, ALTITUDE_<3000_ and AGE_25–65_).

1 $$ln \left(\frac{p\left(HAPE\right)}{1-p\left(HAPE\right)}\right) =-43.69+1.78*{SEX}_{male}+0.66*MCHC+15.93*{ALTITUDE }_{>3000}+2.21*{AGE}_{0-13}+ 1.75*{AGE}_{14-25}+ 21.18*{AGE}_{>65}$$

The objective of the GLM analysis was to find an equation that best predicted the odds of HAPE occurrence and its association with the variables observed in the Eq. 2. Such an equation may provide information about their attributable fraction or *weight* and the deviance linked to any factor of interest. The analysis was only conducted on those significant variables that had an annotated *p* value lower than 0.05 in the ANOVA of the models (data not shown). The variables statistically significant (*p* < 0.05) were Sex, Altitude, MCHC and Age for the fit model (Table 1). The residence below 3000 m.a.s.l was inversely related to the likelihood of having HAPE. Nevertheless, given the influence of the other demographic variables, the multiple logistic regression analyses reflected the dependence of HAPE susceptibility on age. Female sex as well as age were negatively related to the likelihood of having HAPE, whereas the indigenous ethnicity was positively related, yet no statistically significant. The relative influences on HAPE risk were as follows: MCHC > Sex > Altitude > Age _(≥65)_ ≃ Age _(0–13)_ > Age _(14–25)_ ≃ Age _(26–64)_.

The analysis of deviance indicates the relevance of Sex as a risk factor for HAPE in highlands residents and the MCHC as a consistent haematological biomarker for predictions of this condition. In Fig. 2, the black reference lines were added based on an arbitrary threshold odds ratio of 1.05 and considering values over this threshold as significant for MCHC increment. A MCHC value of 34 g/dL was adopted as the reference to analyse the behaviour and correlation for the subjects under study. The latter suggests that a linear increment of MCHC may result in an exponential increase in the risk of HAPE.

## Discussion

Using a statistical analysis that allowed the exploration of the relative influence of the biomarkers of HAPE, it was revealed that, besides the well-known influence of altitude, age and sex, MCHC appeared as the most influential variable in developing HAPE. From a logistic regression analysis, a high concentration of HGB within erythrocytes may predispose to HAPE. To the best of our knowledge, this is the first report identifying MCHC as a blood biomarker for HAPE in Andean populations.

The progress of HAPE is regarded as environmentally mediated by the ascent rate, altitude attained, and prior stay at high altitude^4,20^. However, HAPE is a multifactorial syndrome whose susceptibility presents strong inter-individual differences^20^ that cannot easily be predicted by just the exposure to environmentally-induced hypoxia at high altitude. As expected, some demographic factors like sex and age were inferred from our study. Women were less susceptible to HAPE, and have a better adaptation to high altitude than men^21^. Yet the role of sex in the susceptibility to HAPE has been controversial^4,22^, women actually manage hypoxic ventilation better^23^ and have less erythropoietin and HGB than men, perhaps because of the influence of oestrogen^24^. Age constituted a risk factor for HAPE^25^. Vulnerability to HAPE was high in childhood, decreased in adulthood likely as a result of frequent stays at high altitude and the risk for HAPE increases again in elderly age because of the decline in HGB^26^.

In Andean dwellers, erythrocytosis and haemoglobin concentration above normal average are thought to represent an ideal adaptation to high altitude^27–30^. Our observations only pointed to MCHC, as a likely factor predisposing to HAPE. Some hypotheses should be put forward. Increment of MCHC may lead to high internal erythrocyte-viscosity, decreased deformability, thus increasing pulmonary capillary pressure. The generation of excessive capillary pressure or increased flow in better perfused lung regions may in turn cause HAPE^10,12,31^. This hypothesis would conform with the model of uneven blood flow distribution in HAPE-susceptible patients in hypoxia^8–10,32^, in which strikingly the putative effects of HGB and MCHC were not taken into account. The finding that male sex and paediatric age and the age of elders were risk factors for HAPE was interesting because it does nothing but give support to the putative role for individual differences in HGB concentration in the progress of HAPE^33^. Even if the levels of HGB in Andeans increase for physiological adaptation to high altitude-induced hypoxia^29,34^, they could provide no further benefit in O_2_ transport^35^, likely because of not being conveniently distributed in erythrocytes. Andean highlanders of our HAPE cohort suffered from *re-entry* HAPE^20^, which occurred after a stay of one or two weeks at sea level (max. 200 m.a.s.l.) with no adverse health events until they re-ascended to the Andes ridge (3800 m slope) in a 2-h ride by automobile (unpublished observations). Changes in the rheological properties of blood after exposure to high altitude^36^ may account for the phenomenon of “re-entry HAPE”. This may point to a relationship among the rapid O_2_ concentration change, the HGB distribution in erythrocytes (MCHC) and the malfunctioning of the pulmonary system. Finally, the small number of HAPE individuals compared to the total sample size did not demerit our conclusions, since the HAPE rate (7.5%) coincided just with the middle of the estimated prevalence range (0.01–15.5%).

In summary, this retrospective observational analysis demonstrates the complex confluence of some erythrocyte indices in the adaptation to high altitude-induced hypoxia. Particularly, the role of MCHC in developing HAPE warrants future exciting research.

## Methods

### Study zone

This was an observational study based on two medical centres located at different altitudes. One was the Claudio Benati Hospital in Zumbahua (Ecuador), a remote Andean town sited between 3500 and 4000 m.a.s.l., which approximately serves 20,000 local dwellers (the closest hospital is 64 km away). The second centre was the medical office of the Yachay Tech University (UITEY) in Urcuquí (Ecuador), sited at 2,037 m.a.s.l. in the middle of the Andean corridor (21 km away from the main referral hospital).

### Population description

The group living above 3000 m.a.s.l. consisted of permanent residents of Zumbahua (Ecuador) admitted at the Claudio Benatí hospital (N = 515 subjects). With respect to the group of residents below 3000 m.a.s.l. consisted of both members of the UTEY community entering the university medical office and visitors travelling and sojourning in Zumbahua, who had to seek medical assistance in the Claudio Benatí hospital during their trip (N = 256 subjects). The merged information describing anthropomorphically the cohort studied is presented in Table 2.

**Table 2:** Anthropomorphic description of the cohort under study. Data represents the mean and standard deviation for every collapsed variable.

Altitude Sex Age	BP_Sys		BP_Dias		BMI		Weight (kg)		Height (m)		MCHC (g/dL)		RCB (10^12^/L)		HGB (g/dL)	
	Mean	SD	Mean	SD	Mean	SD	Mean	SD	Mean	SD	Mean	SD	Mean	SD	Mean	SD
< 3000	125.62	26.97	79.21	14.75	23.90	6.73	54.13	24.10	1.47	0.24	32.83	1.28	5.52	0.70	15.61	2.38
F	120.99	24.26	78.32	14.25	24.92	7.23	54.42	22.46	1.46	0.22	32.64	1.33	5.27	0.61	15.03	2.11
> 65	134.33	30.18	82.73	13.47	31.33	3.02	68.33	8.88	1.45	0.08	31.88	0.53	5.81	0.46	16.21	1.06
0–14	100.33	13.91	67.89	13.88	17.70	5.61	26.65	18.45	1.15	0.28	32.53	1.61	5.16	0.47	14.30	1.57
15–24	105.33	12.20	71.63	12.15	24.53	7.79	58.64	18.64	1.57	0.05	32.88	0.93	4.85	0.45	15.13	1.43
25–65	126.01	23.17	80.93	13.97	27.28	5.60	65.50	12.53	1.56	0.08	32.86	1.50	5.29	0.67	14.96	2.50
M	131.92	29.25	80.45	15.41	22.84	6.03	53.84	25.79	1.49	0.26	32.89	1.26	5.59	0.71	16.00	2.48
> 65	156.69	36.94	80.38	17.72	26.23	2.28	64.64	10.57	1.57	0.09	32.68	0.94	5.95	0.99	17.06	2.90
0–14	119.20	11.01	81.80	7.40	17.16	5.02	24.40	15.08	1.17	0.24	32.93	1.22	5.48	0.72	15.41	2.73
15–24	116.81	19.29	71.81	13.05	23.29	3.55	62.42	9.39	1.64	0.06	32.85	1.07	5.46	0.58	15.62	2.73
25–65	134.98	27.77	85.53	14.73	26.30	5.04	73.43	16.78	1.65	0.07	32.95	1.50	5.68	0.66	16.46	1.83
> 3000	103.92	18.95	67.10	13.15	21.80	5.57	45.34	18.83	1.38	0.25	33.69	1.64	5.26	0.61	14.99	1.73
F	103.46	17.92	66.66	12.76	22.45	5.45	45.71	17.12	1.38	0.22	32.68	1.22	5.17	0.59	14.80	1.73
> 65	109.90	21.79	69.07	13.70	23.33	3.57	47.70	8.51	1.42	0.06	32.90	1.74	5.27	0.38	14.97	1.32
0–14	99.73	23.53	60.77	15.75	15.95	3.82	20.25	12.32	1.07	0.27	33.03	1.28	5.30	0.65	14.92	1.79
15–24	99.34	13.28	64.02	10.57	22.65	3.53	50.19	9.67	1.49	0.08	32.69	1.07	5.15	0.56	14.92	1.63
25–65	103.86	17.42	67.77	12.78	25.65	4.29	55.07	10.59	1.48	0.07	32.43	1.03	5.09	0.65	14.61	1.86
M	105.03	21.26	68.16	14.04	20.35	5.57	44.51	22.26	1.37	0.31	34.32	1.55	5.31	0.61	15.19	1.72
> 65	114.69	29.26	73.24	17.38	22.41	3.09	52.60	7.16	1.55	0.07	34.21	1.55	5.39	0.61	15.46	1.55
0–14	87.65	12.29	55.31	16.02	15.45	2.87	20.44	19.99	1.04	0.26	34.10	1.65	5.38	0.67	15.29	1.87
15–24	105.36	19.19	67.88	10.28	20.88	2.10	52.13	10.81	1.58	0.09	34.71	1.60	5.16	0.67	14.88	1.93
25–65	105.30	17.81	69.34	11.92	24.68	5.57	59.79	10.09	1.57	0.09	34.36	1.44	5.29	0.53	15.14	1.54

BP: Blood pressure; BMI: Body mass index.

### Ethics statement

We carried out non-experimental research with secondary data, anonymized patients, type and design with descriptive characteristics in accordance with the ethical policies established by Ecuadorian legislation (PublicHealth Ministerial (MSP) order of December 31st, 2014. Written informed consent was therefore waived due to the retrospective nature of the study. We followed STROBE guidelines to report this study, which was approved by the Ethics Committee of the Claudio Benatí Hospital and the Academic Board of Trustees of UTEY. The authors declare they had no access to identifying patient information when analysing the data. All methods were performed in accordance with the relevant guidelines and regulations.

### Diagnostics

HAPE diagnosis was performed by qualified medical staff and relied on the examination of the cardinal symptoms including dyspnea at rest, tachypnea, crackles, wheezing; dry cough, weakness, chest tightness, and tachycardia^4^, as well as on chest X-ray exams showing central interstitial edema with peribronchial cuffing, ill-defined vessels, and a patchy pattern of airspace consolidation. Patchy opacities produced by HAPE disappeared within days after receiving antidiuretic treatment.

### Data collection and categorization

The database was extracted from patient files dated from the years 2007–2018, and kept in logbooks by authorized medical staff, conforming to a non-probabilistic sampling. Consistency between physical and digital records was meticulously checked to avoid transcription errors. Only demographic, red blood cell parameters and health variables completed in 100% of the patients were included in the analysis (initial data frames for patients living above and below 3000 m.a.s.l were N = 1280 and N = 546 respectively). The terms of the study were defined as follows:

#### Demographic variables

Ethnicity was categorized as indigenes (largely Aymara pedigree) or mestizo (mixed-race like most of the Ecuadorians); sex as male or female; and age classified in the paediatric group (0–13 years), young group (14–25 years), middle age-adult group (26–64 years) and elderly group (≥ 65 years) according to the age classification of the United Nations.

#### Environmental variables

Permanent residence either over or below 3000 m.a.s.l.

#### Health-status (vital signs) variables

Systemic blood pressures, heart rhythm, breathing rate, and blood O_2_ saturation.

#### Erythrocyte indices

Counts of red blood cells (RCB, 10^12^ *L), haematocrit (HCT, %); concentration of haemoglobin (HGB, g/dL), mean corpuscular volume (MCV, fL), mean corpuscular haemoglobin (MCH, pg), and mean corpuscular haemoglobin concentration (MCHC, g/dL). Hematological analysis was performed using a Sysmex XN-550™ Hematology Analyzer (Sysmex America Inc., USA).

### Statistics

All the analyses were carried out using R (R Core Team (2020)), descriptive analysis of data expressing results as mean ± standard deviation (SD) and/or coefficient of variation (CV) was applied. Further comparisons of two variables were based on ANOVA. To understand the behaviour of the HAPE odds and the factors under scrutiny, several Generalised Linear Models (GLM) based on logit linking function^18,19^ were fitted to data to thus determine the relationship between HAPE (0 = non-presence; 1 = presence) (Y) and the explanatory variables (X´s matrix) based on the Eq. 1. These models differed in terms of their explanatory variables (starting with the full model and comparing them until the null model). The probability distribution of the residual error of the model and the estimation method used (frequentist) based on Maximum Likelihood (ML) were used in the selection of the model. Particularly, GLMs were evaluated using the *nlme* (Linear and Nonlinear Mixed Effects Models) package. Furthermore, ANOVA was applied as a post hoc test to models fitted. The criterions applied to select the best model were: (i) simplicity, the model choice is always the simplest model that has the lower Akaike information criterion (AIC), and Bayesian information criterion (BIC); (ii) Likelihood ratio and p-value, considering the probability and ratio that indicates the similarity.

Equation 1. GLM model for the HAPE odds analysis.

2 $$ln \left(\frac{p\left(HAPE\right)}{1-p\left(HAPE\right)}\right) ={B}_{0}+{B}_{1}*SEX+{B}_{2}*MCHC+{B}_{3}*ALTITUDE+\dots +e$$

The Analysis of the goodness of fit for the selected model was based on the relationship between null deviance and the residuals deviance tested by Chi-squared test as difference of deviances. The analysis of deviance based on logit GLM was applied to test the significance of the variables considered in the model and to understand the significance level associated with the deviance of residuals. For all the statistical analysis the significance level was settled at α = 0.05.

### Ethics approval

Study approved by the CBH Ethics Committee and the UTEY Board of Trustees.

## Data Availability

Data will be available on reasonable request. For data access, please contact Prof. Santiago Ballaz; sballaz@yachaytech.edu.ec.
